# Editorial: Advances in machine learning approaches and technologies for supporting nervous system disease diagnosis

**DOI:** 10.3389/fnhum.2023.1295074

**Published:** 2023-10-13

**Authors:** Pedro Miguel Rodrigues, Bruno Catarino Bispo, Diamantino Freitas, João Alexandre Lobo Marques, João Paulo Teixeira

**Affiliations:** ^1^CBQF—Centro de Biotecnologia e Química Fina, Escola Superior de Biotecnologia, Universidade Católica Portuguesa, Porto, Portugal; ^2^Department of Electrical and Electronic Engineering, Federal University of Santa Catarina, Florianópolis, Brazil; ^3^Department of Electrical and Computer Engineering, Faculty of Engineering, University of Porto, Porto, Portugal; ^4^Laboratory of Applied Neurosciences, University of Saint Joseph, Macao SAR, Macao, China; ^5^CEDRI—Research Centre in Digitalization and Intelligent Robotics and SusTEC—Associate Laboratory for Sustainability and Technology, Instituto Politécnico de Bragança, Bragança, Portugal

**Keywords:** nervous system disease, machine learning, technologies, diagnosis, detection

The nervous system is essential for physical and mental health but is complex and delicate. As it can unfortunately be affected by several progressive diseases, an early diagnosis is often critical for effective treatment (Xu et al., 2022). The diagnosis of nervous system diseases traditionally relies on a combination of clinical examination, imaging and signals tests, and laboratory tests (Siuly and Zhang, 2016). However, these methods can be time-consuming, expensive, and not always accurate (Milligan, 2019).

In an era marked by unprecedented technological advances in machine learning (ML), a computational tool that allows the identification of patterns in data that would be difficult or even impossible for humans, its application to assist in medical diagnosis emerges as a beacon of hope in the complex panorama of nervous system diseases. The Research Topic *Advances in machine learning approaches and technologies for supporting nervous system disease diagnosis* aims to shed light on the transformative role that ML-based approaches and technologies are playing in reshaping the way an ensemble of nervous system disorders are understood, diagnosed, and treated.

The pursuit of biomarkers that illuminate disease progression and treatment response has been a long-standing goal. The analytical prowess of ML has invigorated this quest by rapidly sifting through voluminous datasets to uncover novel biomarker candidates (Valliani et al., 2019). This acceleration in discovery has the potential to unlock diagnostic insights that traditional methodologies might have overlooked. By categorizing conditions like Alzheimer's (Rodrigues et al., 2021; Kavitha et al., 2022; Silva et al., 2023), Parkinson's (Makarious et al., 2022; Silva et al., 2022), and Epilepsy (Jin et al., 2020; Brari and Belghith, 2021) with unprecedented accuracy, ML is charting a more confident course toward differentiating disease subtypes and stages.

This Research Topic contributes to the area with the following studies:

- Liang et al. investigated trigeminal neuralgia (TN), which is a severe facial pain condition with unknown causes and variable treatment responses. Functional magnetic resonance imaging (MRI) data from TN patients during resting and pain-tracking sessions were used. Three analysis methods (conventional, CNN, and GCNN) identified common pain-related brain regions: the superior temporal cortex, insula, fusiform, precentral gyrus, superior frontal gyrus, and supramarginal gyrus. Additionally, 17 regions, such as the dACC and thalamus, were identified using CNN and GCNN methods with *R* = 0.62 and 0.60, respectively, both with a *p*-value < 2 × 10^−3^. It was concluded that these 23 regions are key centers of TN pain, shedding light on this topic for future research.
- Gruss et al. investigated the ability of electromyography (EMG) to, in a postoperative setting, distinguish between pre- and post-analgesic administration to assess pain in non-verbal patients. Biosignals from 38 patients were recorded before and after receiving analgesic sedation. The results showed that EMG can distinguish between pre- and post-analgesic administration with a high degree of certainty (*R* = 0.56).
- AlSharabi et al. explored the potential of electroencephalography (EEG) data for the early detection of Alzheimer's disease (AD) to develop a reliable clinical decision support system. The study applied bandpass filtering and empirical mode decomposition to extract features in EEG signals from neurotypical individuals and AD patients. By employing various ML techniques, classification accuracies ranging from 94.8 to 99.9% were achieved. These findings underscored the promising role of EEG-based diagnostic support as a supplementary tool for early AD diagnosis, with implications for diagnostic biomarker identification.
- Sun et al. used preoperative arterial spin labeling MRI to investigate deep gray matter perfusion in neonates with congenital heart disease (CHD). Results showed lower right thalamus perfusion in cyanotic CHD vs. controls and lower right basal ganglia perfusion in cyanotic CHD vs. acyanotic CHD. The CHD group collectively exhibited a slight decrease in left thalamus perfusion compared with healthy controls. These findings highlight the influence of cardiac physiology on regional cerebral perfusion changes and suggest arterial spin labeling as a potential marker for identifying high-risk cases of cerebral blood flow dysregulation and hypoperfusion in neonatal CHD.
- Shi et al. proposed a novel dynamic functional connectivity (dFC) analysis method for functional MRI studies, applied to schizophrenia research. By exploring multiple frequency bands and abnormal regions of interest identified using the fractional amplitude of low-frequency fluctuations, the method improved the classification of schizophrenia patients compared with conventional approaches, achieving an accuracy of 91.21% using an SVM classifier. Sliding time window analysis and feature selection further improved the performance. These contributions propel research on brain alterations in schizophrenia toward uncharted healthcare territories.

In conclusion, the fusion of ML with nervous system disease diagnosis is transforming healthcare profoundly. This journey into uncharted territory holds the promise of elevating diagnostic precision and redefining the understanding of these disorders. The application of ML to neuroimaging has been shown to bring profound insights, including the discovery of novel biomarkers. Collaboration between the medical community, data scientists, and ethicists is essential. Together, they can harness the potential of ML in improving diagnostic outcomes and enhancing patient quality of life, ushering in an era of enriched healthcare through the synergy of technology and medicine.

## Author contributions

PR: Conceptualization, Investigation, Supervision, Validation, Writing—original draft, Writing—review and editing. BB: Investigation, Validation, Writing—review and editing. DF: Writing—review and editing. JL: Writing—review and editing. JT: Writing—review and editing.
